# Effects of Creatine Monohydrate Loading on Sleep Metrics, Physical Performance, Cognitive Function, and Recovery in Physically Active Men: A Randomized, Double-Blind, Placebo-Controlled, Crossover Trial

**DOI:** 10.3390/nu17243831

**Published:** 2025-12-07

**Authors:** Khouloud Ben Maaoui, Slaheddine Delleli, Nourhène Mahdi, Arwa Jebabli, Juan Del Coso, Hamdi Chtourou, Luca Paolo Ardigò, Ibrahim Ouergui

**Affiliations:** 1 High Institute of Sport and Physical Education of Sfax, University of Sfax, Sfax 3000, Tunisia; benmaaouikhouloud88@gmail.com (K.B.M.); sdelleli2018@gmail.com (S.D.); nourhene648@gmail.com (N.M.); jebabliarwa@gmail.com (A.J.); h_chtourou@yahoo.fr (H.C.); 2 Research Laboratory, Education, Motricity, Sport and Health, EM2S, LR19JS01, University of Sfax, Sfax 3000, Tunisia; 3 Physical Activity, Sport and Health, Research Unit, UR18JS01, National Sport Observatory, Tunis 1003, Tunisia; 4 Sport Sciences Research Centre, Rey Juan Carlos University, 28943 Fuenlabrada, Spain; juan.delcoso@urjc.es; 5 Department of Teacher Education, NLA University College, 0166 Oslo, Norway; 6 High Institute of Sport and Physical Education of Kef, University of Jendouba, El Kef 7100, Tunisia; 7 Research Unit, Sport Sciences, Health and Movement, UR22JS01, University of Jendouba, El Kef 7100, Tunisia

**Keywords:** nutritional supplements, sleep quality, ergogenic aids, exercise, sports performance

## Abstract

**Background/Objectives:** Creatine monohydrate (CrM) supplementation is well-established for enhancing physical performance and accelerating recovery in several sporting contexts. However, beyond these traditional performance benefits, its effects on sleep metrics and cognitive function have not been thoroughly investigated. This investigation aimed to determine the effect of a loading phase of CrM on sleep metrics, physical performance, psycho-cognitive aspects, and recovery in physically active men. **Methods**: In a randomized, double-blind, placebo-controlled crossover design, 14 physically active men ingested 20 g/day of CrM or placebo (PL) for 7 days, during which their habitual exercise routines were maintained and standardized across both intervention phases. Sleep metrics were monitored throughout the interventions using wrist-worn actigraphy. On the day following the completion of each supplementation phase, participants rated their sleep quality using the Sleep Subjective Quality (SSQ) scale, and the Hooper questionnaire was used to monitor participants’ well-being status. Physical performance was assessed using the 5 m shuttle run test (5mSRT), which measured total distance (TD), best distance (BD), performance decrement (PD), fatigue index (FI), and the rating of perceived exertion (RPE). Affective valence was determined using the feeling scale (FS) and cognitive function was evaluated using the digit cancellation test (DCT). Recovery and muscle soreness perceptions were evaluated at multiple time points (pre-exercise, 5 min, 24 h, 48 h, and 72 h post-exercise) using the perceived recovery status (PRS) and the delayed onset muscle soreness (DOMS) scales, respectively. **Results**: During the supplementation, CrM improved sleep quality compared to PL, as measured with the SSQ scale (*d* = 0.81, *p* = 0.009), and was associated with an earlier in-bed time (r = 0.60; *p* = 0.026). However, CrM did not affect sleep latency (t = 0.98; *p* = 0.35), sleep efficiency (t = 0.018; *p* = 0.98), or total sleep time (t = 0.25; *p* = 0.81). After the supplementation phase, CrM resulted in significantly lower muscle soreness scores, as measured by the Hooper questionnaire (*d* = −0.59; *p* = 0.046), improved cognitive performance on the DCT (*d* = 0.77; *p* = 0.013), and enhanced TD (r = 0.88; *p* < 0.001) and BD (r = 0.76; *p* < 0.05) during the 5mSRT. However, CrM did not significantly affect other exercise-related measures such as RPE, fatigue index (FI), or performance decrement (PD) during the 5mSRT, nor did it alter other subjective recovery scales compared to PL, up to 72 h following the end of the supplementation phase (all *p* > 0.05). **Conclusions**: A 7-day CrM loading protocol improved subjective sleep quality during the supplementation phase, enhanced cognitive performance, and increased physical output during high-intensity intermittent exercise. CrM also reduced muscle soreness, but did not significantly affect objective sleep parameters, or recovery markers up to 72 h post-exercise. These findings suggest that CrM may offer additional benefits beyond its traditional ergogenic role. Trial Registration: This trial was registered on 18 September 2023 at the Pan African Clinical Trials Registry (PACT) (identifier: PACTR202309597156293).

## 1. Introduction

Athletes and physically active individuals are increasingly exploring ergogenic supplements as a strategy to enhance exercise performance [1]. Among the plethora of ergogenic substances used to enhance exercise performance, creatine (Cr) (α-methyl guandino-acetic acid) holds a unique position [2]. The kidneys, liver, and pancreas are the main endogenous organs that produce Cr through a two-step process that involves the amino acids arginine, glycine, and methionine [3]. However, given that intramuscular phosphocreatine (PCr) reserves are limited, athletes and exercisers frequently consume exogenous supplements of Cr, such as creatine monohydrate (CrM), to increase muscle mass, performance, and recovery [4]. Cr ergogenic potential is related to its crucial function in regulating energy metabolism [5]; it has been extensively demonstrated that increasing the dietary intake of Cr leads to elevated levels of Cr and PCr in skeletal muscles [6].

A common misconception about Cr supplementation is that a “loading phase” is essential to achieve the purported ergogenic benefits [4]. Specifically, short-term Cr loading, typically involving a daily intake of 20–25 g for 5 to 7 days, can increase muscle PCr stores to the point of saturation (20–40%), leading to improvements of about 5–10% in high-intensity, short-duration exercise performance [6]. The increase in intramuscular concentrations of PCr leads to a greater reliance on the phosphagen system during intense efforts [7], facilitating adenosine triphosphate (ATP) transport from their sites of production (i.e., mitochondria) to those of utilization [8], which reduces oxidative stress [9]. Moreover, Cr loading has emerged as a promising strategy in mood regulation, offering potential relief for depression symptoms and improving overall emotional well-being [10]. In sport settings, Cr loading not only enhances physical performance but also helps to reduce mental fatigue and improve mood states following exercise, thereby supporting recovery and overall mental resilience [11,12]. This multifaceted role of Cr underscores its value not only for athletes but also for individuals seeking to enhance both cognitive and emotional health.

Although skeletal muscle stores the majority of the body’s creatine, the brain is a highly metabolically active organ, accounting for approximately 20% of total energy consumption [13]. The brain’s substantial energy requirements necessitate efficient replenishment mechanisms [12], which are predominantly active during sleep [14]. Sleep plays a vital role in maintaining human health, primarily by supporting the recovery and regeneration of physiological and psychological systems [15]. This includes the restoration of neurochemical, hormonal, muscular, and immunological functions [14]. Adequate quality and quantity of sleep are essential for maintaining well-being, mood stability, and cognitive performance [15].

Given the brain’s reliance on sleep for energy restoration and neurochemical recovery, creatine supplementation may positively influence cognitive functions. Specifically, Cr metabolically supports processes such as memory consolidation and synaptic plasticity by enhancing ATP recycling, thereby ensuring rapid energy availability during periods of high neural demand [16]. These processes emphasize the significance of Cr as a major contributor to energy metabolism as well as a possible regulator of mood and cognitive function [13,17]. Additionally, when brain bioenergetics, which refers to cellular metabolism and energy homeostasis of the brain, are challenged, as occurs during sleep deprivation, the reliance on Cr as a source of energy seems to be stronger [18,19]. Sleep deprivation, often regarded as a metabolic disorder due to its disruption of energy homeostasis, drives neurons into a catabolic state [19]. This catabolic condition is further exacerbated by the increased metabolic demands of prolonged wakefulness and the concomitant reduction in energy substrate availability [18]. Increased Cr levels in the brain may improve resilience and mental function, reducing cognitive impairments brought on by various stressors [20]. Although the implications of this are yet unclear, it is linked to a decrease in the perceived effort associated with cognitively taxing tasks [20]. However, despite Cr being able to cross the blood–brain barrier (via microcapillary endothelial cells expressing the Cr transporter SLC6A8), its uptake into the brain is less effective than that of skeletal muscle [21]. This suggests that brain Cr levels could be relatively independent of external sources, such as dietary Cr ingestion [22]. Yet, it is unclear how these variations in Cr uptake between the brain and muscles may be explained.

The ergogenic effects of Cr supplementation appear to be particularly pronounced under conditions that challenge cognitive functioning, such as sleep deprivation [12,23], complex cognitive tasks, or hypoxia [24,25]. Studies in sleep-deprived participants have shown that Cr supplementation may attenuate the cognitive and physical impairments associated with sleep loss [23]. Among naturally menstruating females, Cr supplementation increases sleep duration following a resistance training day [26]. While the additive effect of Cr intake on sleep has been coupled with stressful conditions, the impact of Cr loading on sleep metrics and performance-related aspects in normal conditions (i.e., no-stressor factors) remain under-explored. Therefore, this study was designed to evaluate whether CrM loading affects sleep patterns, physical performances, psycho-cognitive responses, and recovery perceptions in physically active individuals. Based on prior evidence supporting the benefits of creatine monohydrate on physical and cognitive performance, as well as recovery, we hypothesized that a short-term loading protocol would positively influence sleep quality, exercise performance, cognitive function, and recovery perceptions in physically active individuals.

## 2. Materials and Methods

### 2.1. Participants

The required sample size was calculated a priori using the G*Power software (Version 3.1.9.7, University of Kiel, Kiel, Germany). Using the *t*-test family for the difference between two dependent means (matched pairs), with α set at 0.05, power (1−β) set at 0.80, and an effect size of 0.94 previously reported for fatigue index following a loading protocol of 0.3 g/kg of CrM during 5 days [27], the analysis revealed that nine participants were required to approach an actual power of 82%. To avoid dropouts during the study, 14 physically active men were recruited to participate in the present study (Mean ± SD; age: 23.86 ± 2.28 years; height: 1.77 ± 5.81 m; body mass: 80.71 ± 7.53 kg). To be eligible, participants had to meet the following criteria: (i) they were non-smokers, (ii) did not present any pathological sleep disorders (i.e., the Pittsburgh Sleep Quality Index was <5 [28]) or insufficient sleep hygiene (using the sleep hygiene index [29]), (iii) did not suffer from injuries, (iv) did not consume alcohol or foods rich in antioxidants/polyphenols, (v) did not use any type of medicine or dietary supplement during the duration of the experiment, (vi) followed an omnivore diet but without consuming Cr supplements, and (vii) participated in a variety of activities and/or sports for 2 or more days a week without a specific commitment or focus on sports competition. After a comprehensive explanation of the study’s procedures, potential benefits, and risks, all participants provided written informed consent. The study was conducted in accordance with the Declarations of Helsinki, and the protocol was fully approved by the Committee of Protection of Southern Persons (CPP: N° 0414/2022) (approval date: 26 August 2025).

**Figure 1:**
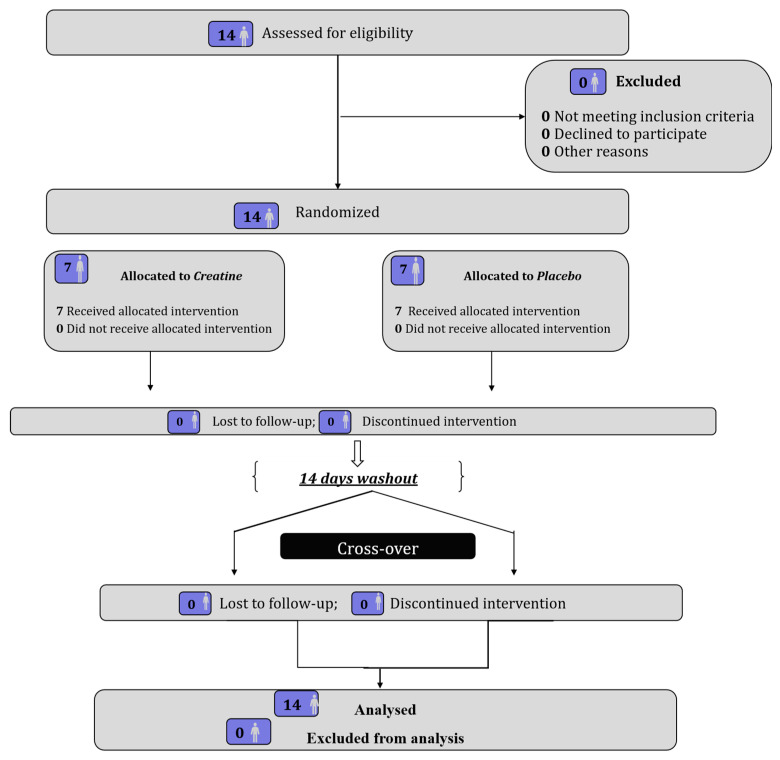
Participants flow diagram.

The participants flow diagram is presented in Figure 1.

### 2.2. Experimental Design

**Figure 2:**
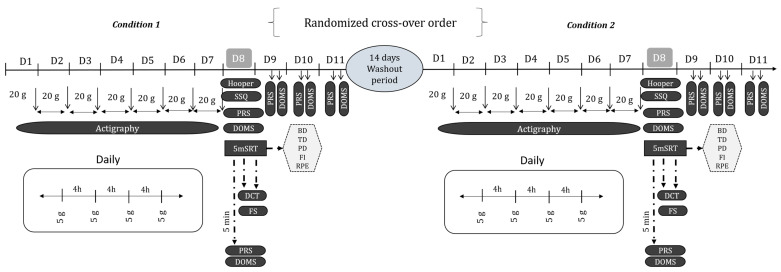
Schematic representation of the study design; D: day; DCT: digit cancellation test; SSQ: sleep subjective quality; FS: feeling scale; PRS: perceived recovery status; DOMS: delayed onset muscle soreness; 5mSRT: 5 m shuttle run test: BD: best distance; TD: total distance; PD: performance decrement; FI: fatigue index; RPE: rating of perceived exertion.

The current experiment was a randomized double-blind, placebo-controlled, crossover study that followed the Consolidated Standards of Reporting Trials (CONSORT) guidelines for a randomized crossover trial [30] (Supplementary Table S1). The study was designed to determine the effects of CrM supplementation (i.e., only the loading phase) on sleep metrics, physical performance, psycho-cognitive responses, and recovery perception in healthy males. For each participant, the total duration of the experiment was >28 days, including the familiarization, the two intervention periods, and the washout period between treatments. To complete the experiment, participants visited the laboratory on three occasions. During the first visit, participants were familiarized with the testing procedures and height, and body mass measurements were performed. Following familiarization, and in a randomized order, participants underwent a 7-day loading phase, during which they consumed 20 g/d of either creatine monohydrate (CrM; Powder Pro Zero) or placebo (PL; corn-starch maltodextrin), divided into four equal servings of 5 g each. This dosage was selected based on prior evidence indicating that daily intakes of 20 g or more reliably increase creatine stores in healthy individuals [31]. A four-hour interval was granted between servings, since orally ingested CrM (e.g., 5 g/day) increases blood concentrations of Cr for 3–4 h after ingestion, thereby facilitating the uptake of Cr into tissue [3]. During each day of the loading period with either CrM or placebo, participants were equipped with the ActiGraph GT3 activity monitor (ActiGraph TM Corp., Pensacola, FL, USA). The activity monitor was worn on the non-dominant wrist to capture daily sleep–wake data and to monitor participants’ adherence to a regular sleep schedule [32]. Additionally, participants maintained their habitual diet and normal sleep practices for the duration of the experimental period. To avoid identification and assure blinding success, supplements were administered in opaque, unmarked containers and handled by an independent person. Each serving of CrM or PL (i.e., 5 g) was administered in powder form dissolved in 500 mL of distillated water at room temperature (~28 °C) to ensure optimal solubility. This was enough to entirely dissolve the 5 g, since 6 g of Cr dissolves in one liter of water at 4 °C [33]. To preserve blinding and maintain experimental control, participants were instructed to refrain from discussing or comparing the taste of the beverages or speculating about their content. Investigators directly supervised ingestion to ensure full consumption of each dose and to prevent any exchanges between participants. During the testing days (i.e., the day after the end of each loading phase), the session started by rating the Hooper Questionnaire to monitor well-being status [34]. Moreover, sleep quality was evaluated using the Sleep Subjective Quality (SSQ) questionnaire. Then, participants completed 10 min of standardized warm-up consisting of light runs and dynamic stretching. Following this, participants completed the 5 m shuttle run test (5mSRT), in accordance with the standardized procedure reported previously [35]. After every repetition of the 5 m shuttle run test, participants reported their perceived exertion using the Borg CR-10 scale [36]. After test completion, the digit cancellation test (DCT) was rated to assess participants’ attention and alertness [37] and the feeling scale (FS) was used to evaluate their general emotional valence [38]. In addition, scores for self-perceived recovery (PRS) [39] and delayed onset muscle soreness (DOMS) [40] were assessed at multiple time points (i.e., pre-testing and 5 min, 24 h, 48 h, and 72 h post-exercise). All psychometrics outcomes (i.e., Hooper, SSQ, RPE, PRS, DOMS) were analyzed as raw scores obtained directly from participants’ responses on validated questionnaires. To ensure a sufficient period for Cr elimination, conditions were separated by 14 days of washout [41] (Figure 2). Participants were randomized using a structured block-randomization procedure with rank-based allocation to ensure balanced group sizes and to minimize allocation bias. Specifically, blocks of seven participants were created, and within each block, a random number was assigned to each participant using the RAND () function in Microsoft Excel 2007 (Redmond, WA, USA). Participants were then ranked in ascending order based on these random numbers. The treatment condition was allocated according to rank, with odd-ranked participants assigned to the CrM condition and even-ranked participants assigned to the PL condition. To reduce the influence of the circadian rhythms on the measured outcomes, the testing sessions were scheduled during the morning hours.

### 2.3. Measures

#### 2.3.1. Actigraphy

Actigraphy consisted of measuring body movement during sleeping using motion sensors, commonly worn on the non-dominant wrist [28]. Participants were instructed to wear the actigraphy for seven days and the device was set to record movement data at 1 min intervals. They were instructed to press the event marker button on the actigraphy to digitally mark times they went to bed and times they got out of bed. Participants also kept a sleep diary for each day where they recorded ‘in-bed times’, ‘get up times’, and the times the actigraphy was removed from the wrist (i.e., non-wear periods or off-wrist periods) [42]. Although the software generates about two dozen sleep parameters, the variables considered for analysis were the in-bed time (the time point when the subject first lie down in-bed), out-bed time (when the subject finally arose or got out of bed), total minute in bed (refers to the duration a participant spent in bed and is derived by subtracting the in bed time from the out-bed time), total sleep time (the duration of the interval from sleep onset to sleep offset), efficiency (percentage of time spent asleep during the total sleep time) and latency (the amount of time it takes to fall asleep). The value of each sleep parameter is calculated separately for each 24 h period (i.e., 1 day) and then the estimates across the multiple days are averaged to obtain a more stable/representative measure that minimize inter-daily variability [43].

#### 2.3.2. The Hooper Questionnaire

The Hooper questionnaire is composed by four items (i.e., stress, sleep, fatigue and delayed onset muscle soreness) [34]. Each item was scored on a seven-point Likert scale, with answers ranging from “very, very good” to “very, very bad” for sleep, and “very, very low” to “very, very high” for fatigue, stress, and muscle soreness. The total hooper index (HI) was determined using the sum of the four items. The questionnaire was rated at the beginning of each testing session to assess the well-being status of each participant following the loading period.

#### 2.3.3. Subjective Sleep Quality

Subjective ratings of sleep quality were assessed using the SSQ scale of 0–10, where 0 indicated “no sleep”, 5 indicated “some sleep with some interruptions”, and 10 indicated “uninterrupted, deep sleep throughout” [44]. The questionnaire was rated at the beginning of each testing session to assess the sleep quality of each participant pre-testing.

#### 2.3.4. 5m Shuttle Run Test

The 5mSRT is a high-intensity, intermittent sprint test designed to assess anaerobic capacity, agility, and repeated sprint ability. It mimics the demands of several sports by incorporating short, maximal-effort sprints and frequent changes in direction. It consists of six repetitions of 30 s shuttle sprints, as previously described [35]. The distance increases from one repeat to the next as the runner dashes back and forth over a predetermined shuttle distance. There is a passive recovery phase of 35 s after every shuttle run bout. A level surface with two parallel lines spaced five meters apart is used for the test. For each repetition, the athlete sprints 5 m to the opposite line, touches it with one foot executing a sharp 180-degree turn, and sprints back to the starting line. The distance completed in each repetition was measured, and both the best distance (BD) obtained in a single 30 s shuttle and the total distance (TD) over the six shuttles were documented. Additionally, the fatigue index (FI) and percentage decrement (PD) [35] were calculated as follows:

TD (m) = sum of distances covered during the 6 × 30 s shuttles

BD (m) = highest distance covered during one of the 6 × 30 s shuttles

(1) $$FI\left(\%\right)=\frac{\left[\frac{shuttle\;1\;+\;shuttle\;2}{2}\;-\;\frac{shuttle\;5\;+\;shuttle\;6}{2}\right]}{\frac{shuttle\;1\;+\;shuttle\;2}{2}}\times 100$$

(2) $$PD\left(\%\right)=\frac{\left[\left(BD\times number\;of\;sprints\right)\;-\;TD\right]}{\left(BD\times number\;of\;sprints\right)}\times 100$$

#### 2.3.5. Rating of Perceived Exertion

Perceived exertion was determined using the Borg CR-10 scale, which ranges from 0 to 10, with descriptors indicating progressively greater intensity of effort (0 = nothing at all; 10 = extremely strong) [36]. The mean RPE value for the whole test, calculated from the scores obtained after each 5mSRT repetition/bout, was used for statistical analysis according to the following formula [45]:

RPE (a.u) = Σ (RPE scores)/number of repetitions(3)

#### 2.3.6. The Digit Cancellation Test

The DCT measures an individual’s ability to focus, scan information, and sustain attention over a period of time. It consists of four pages, each one containing 600 numbers of one to five digits, spread over 36 lines [37]. It was developed to assess various aspects of the prefrontal cortex performance, such as attention, information processing speed, and executive functioning [37]. Participants had to erase as many three-digit numbers as possible for 1 min. The number of targets was 187, with two to eight three-digit numbers randomly distributed per line. The numbers were separated by a point and preceded and followed by a space. The total number of targets correctly erased represents an assessment of the athlete’s vigilance [37].

#### 2.3.7. Feelings Scale

The FS is a tool proposed to evaluate emotional valence [38]. The FS consists of a 10-point bipolar scale ranging from +5 to −5, with verbal associations of very good (+5), good (+3), moderately good (+1), neutral (0), just as bad (−1), bad (−3), and very bad (−5) [38].

#### 2.3.8. Perceived Recovery Scale

An 11-point scale was employed to determine participants’ recovery state, where participants were assigned scores from 0, representing very low recovery or extreme tiredness, to 10, reflecting very good recovery or high energy levels [39]. The PRS scale was rated on paper pre-testing, and at different time points after exercise (i.e., 5 min, 24 h, 48 h, and 72 h).

#### 2.3.9. Delayed Onset Muscle Soreness

Delayed onset muscle soreness (DOMS) was assessed using a 10-point scale, where 1 indicated no pain and 10 represented extreme pain, reflecting the participant’s subjective perception of lower-limb muscle soreness [40]. The scale was completed on paper prior to exercise testing and again at 5 min, 24 h, 48 h, and 72 h post-exercise.

### 2.4. Statistical Analyses

The statistical analysis was performed using SPSS 27.0 statistical software (IBM corps, Armonk, NY, USA). The Shapiro–Wilk test was used to check and confirm the normality of data sets. Data are presented as mean (SD) for normally distributed variables and as median (MED)/interquartile (IQR) for non-normally distributed ones. The Levene test was used to verify the homogeneity of variances. Sphericity was tested using the Mauchly test. Actigraphy-derived data were exported in time format (hh:mm:ss) into an Excel spreadsheet. They were then converted to minutes using decimal values and subsequently imported into SPSS for analysis as continuous variables. For variables with normal distribution, the student *t*-test for dependent samples was performed to assess differences between conditions (CrM vs. PL). Standardized effect size analysis (Cohen’s *d*) was used to interpret the magnitude of differences between variables and considered as trivial (<0.20); small (<0.50); moderate (0.50–<0.80); large (<1.20); and very large (>1.20) [46]. Moreover, the 95% confidence interval of the difference (95%CI_diff_) was calculated. For variables with non-normal distribution, the Wilcoxon signed rank test for matched pairs was used to compare conditions and the Friedman test was used for variables measured at different time points (i.e., PRS and DOMS). To assess the magnitude of the differences, the rank biserial correlation coefficient (r) was calculated using the Wilcoxon Z-scores and the total number of observations (N) (i.e., r = Z/√N) and considered as 0.1 to <0.3 (small), 0.3 to <0.5 (moderate), and ≥0.5 (large) [47]. Significance was set at *p* < 0.050 for all analyses.

## 3. Results

### 3.1. Actigraphy Variables

The Wilcoxon test showed that CrM advanced the in-bed time compared to PL (Z = −2.23; r = 0.60; *p* = 0.026). However, the *t*-test showed no difference between CrM and PL in terms of the out of bed time (t = 1.26; *p* = 0.23), sleep latency (t = 0.98; *p* = 0.35), sleep efficiency (t = 0.018; *p* = 0.98), total minutes in bed (t = 0.53; *p* = 0.60), and total sleep time (t = 0.25; *p* = 0.81) (Table 1).

### 3.2. Hooper Questionnaire

The *t*-test showed a significant difference in muscle soreness between conditions (t = −2.20; *p* = 0.046), with lower values under CrM compared to PL (95%CI_diff_: −2.70 to −0.03; *d* = −0.59). However, there was no effect on fatigue (t = −0.61; *p* = 0.55) and HI (t = 0.50; *p* = 0.63). Similarly, the Wilcoxon test showed no effect on sleep (Z = −0.80; *p* = 0.43) or stress (Z = −1.21; *p* = 0.23) (Table 2).

### 3.3. Sleep Subjective Quality

Regarding SSQ, the *t*-test showed a main effect of condition (t = 3.05; *p* = 0.009), with higher values recorded under CrM supplementation compared to PL (95%CI_diff_: 0.54 to 3.17; *d* = 0.81) (Table 2).

### 3.4. Physical Performance and Perceived Exertion

For TD, the Wilcoxon test showed higher values after CrM supplementation than with PL (Z = −3.30; r = 0.88; *p* < 0.001). For BD, values after CrM ingestion were higher compared to PL (Z = −2.84; r = 0.76; *p* < 0.05). However, the *t*-test showed no significant difference between conditions for FI (t = 0.56; *p* = 0.58), PD (t = 1.50; *p* = 0.16) and RPE (t = 0.84; *p* = 0.42) (Table 3).

### 3.5. Digit Cancellation Test

The *t*-test showed a significant difference in the DCT scores between conditions (t = −2.88; *p* = 0.013), with higher values recorded under CrM compared to PL (95%CI_diff_: 0.16 to 1.36; *d* = 0.77) (Table 4).

### 3.6. Feeling Scale

The Wilcoxon test showed no difference in FS scores between conditions (Z = −0.99; *p* = 0.32) (Table 4).

### 3.7. Recovery Perceptions

#### 3.7.1. Delayed Onset Muscle Soreness

**Figure 3:**
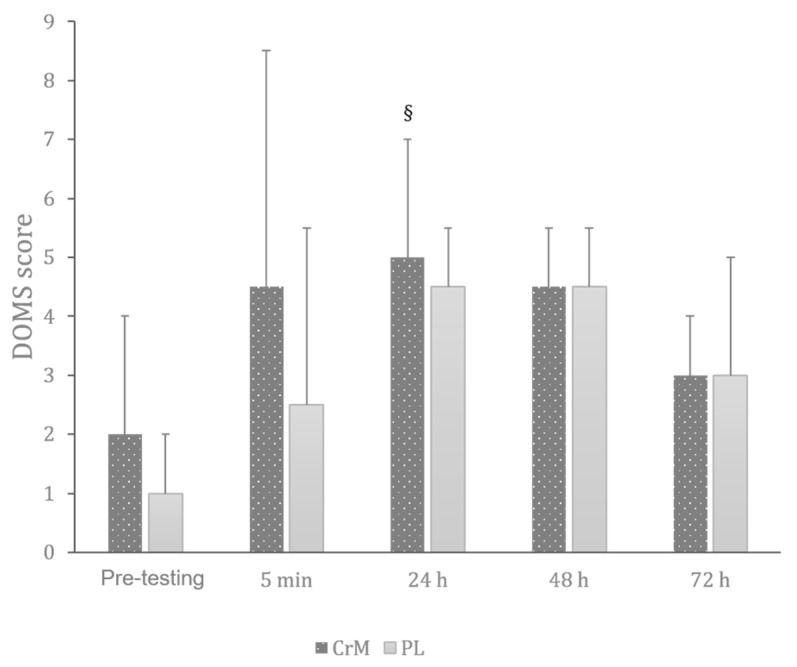
Variation in the DOMS scores across creatine monohydrate (CrM) and placebo (PL) conditions at different times of measurement. §: significantly higher than PL at the same time point (*p* < 0.05).

The Friedman test revealed a significant effect of time on DOMS across conditions (Chi2 = 68.66; df = 9; *p* < 0.001). As presented in Figure 3, the Wilcoxon test revealed that CrM elicited significant higher DOMS values than PL 24 h after the exercise (Z = −2.41; r = −0.64; *p* = 0.016), with no other differences between conditions in the other time points.

#### 3.7.2. Perceived Recovery Status

**Figure 4:**
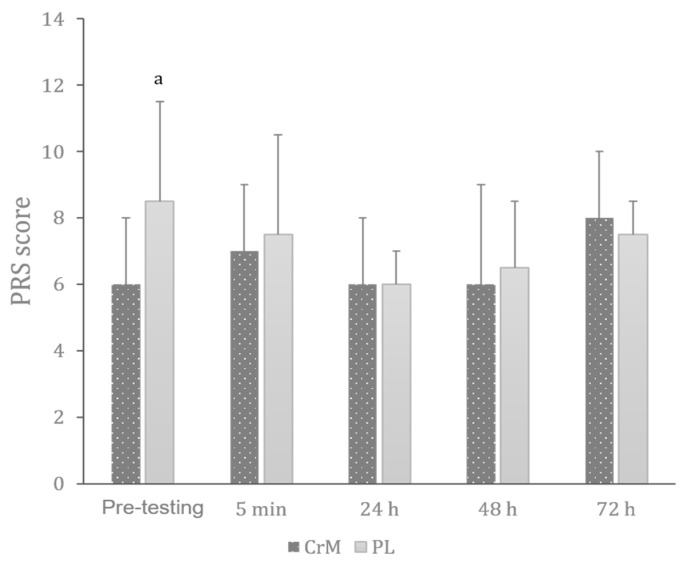
Variation in the perceived recovery status (PRS) scores across creatine monohydrate (CrM) and placebo (PL) conditions at different times of measurement. a: significantly higher than CrM at the same time point (*p* < 0.05).

The Friedman test revealed a significant effect of time on PRS across conditions (Chi2 = 34.93; df = 9; *p* < 0.001). As presented in Figure 4, CrM elicited lower values than PL only in pre-testing (Z = −2.41; r = −0.64; *p* = 0.016), with no other differences between conditions in the other time points.

## 4. Discussion

The present study investigated the effect of CrM loading on sleep metrics, physical performance, cognitive function, psychological states, and recovery perception in physically active men. Compared to PL, 7 days of CrM intake advanced the in-bed time during the loading period while improved SSQ and reduced muscle soreness sub-scale after CrM also enhanced TD and BD during the 5 mSRT and increased the DCT scores.

Adequate quality and quantity of sleep are essential for both physiological and psychological systems’ regeneration [15]. In the present study, CrM loading improved sleep quality, and advanced the in-bed time without affecting sleep quantity. Cr acts like a temporal and spatial high-energy phosphate-storage buffer [8], enhancing energy availability in the brain and potentially supporting more efficient brain function [48]. Particularly, during states of heightened metabolic demand such as during sleep loss, when the brain undergoes intensive restorative processes, Cr serves as a vital energy reservoir [12]. In fact, it has been reported that 7 days of CrM supplementation increased brain Cr content by 9.2% [25]. This energy boost can facilitate critical processes like neurotransmitter synthesis (e.g., serotonin and dopamine), neuronal activity, and brain plasticity, all of which are essential for managing stress and regulating emotions [17]. These effects may lead to a more stable neurological environment [16], positively influencing sleep architecture and overall sleep quality.

Regarding sleep quantity and actigraphy parameters, the effects of CrM are often more notable under stress conditions, such as sleep disruptions [12,23]. Since the recruited individuals in the present study typically have adequate sleep hygiene and did not present any pathological sleep disorders, CrM supplementation did not lead to noticeable improvements. Moreover, the total amount of CrM ingestion may need to be higher or taken for longer periods of time to induce significant effects in the brain compared to skeletal muscle [13]. This is related to the higher permeability of muscular tissues to uptake the Cr circulating in the blood (i.e., ~95% is stored in skeletal muscle) compared to the brain [49]. From another perspective, as in muscle, the brain has the ability to synthesize Cr [12,50] with lower storage capacity [22], which may contribute to its relative resistance to exogenic CrM uptake [13].

Evidence indicates that CrM supplementation can enhance performance in response to intense exercise and enables athletes to sustain higher levels of maximal effort over repeated bouts [2]. The current study reported improved physical performance in response to acute CrM administration in young healthy men. The present findings corroborate earlier studies reporting that acute CrM supplementation enhances power generation and performance during repeated exercise efforts [27,51,52]. However, not all investigations have confirmed these effects [53]. Several studies have reported that brief periods of CrM supplementation improve high-intensity exercise performance, reflected by increases in power output and total work [51,52]. These benefits are thought to result from higher PCr stores in muscle, allowing for more rapid energy turnover during repeated bouts of effort. Notably, muscle creatine levels can reach saturation after only a few days of supplementation [54]. Particularly, short-term or long-term supplementation with CrM increases muscle PCr reserves by 20–40%, amplifying, consequently, the effects of this energy source on physical performance [6]. CrM supplementation may boost and sustain the flow of ATP to working muscles, enabling enhanced efficiency [6]. Increasing ATP resynthesis provides more accessible energy during exercise, enlarging the skeletal muscles’ work capacity, postponing the start of muscle exhaustion, and enhancing performance [1]. In this regard, it was discovered that consuming CrM reduced ATP metabolism by around 30%, and this sustainment persisted even after performing further exercise [52]. These findings are significant because they demonstrate the crucial connection between intramuscular PCr status and the decline in exercise performance [1]. Alternatively, CrM supplementation may influence exercise performance by boosting myofibrillar cross-bridge cycling and force generation through increased calcium re-uptake into the sarcoplasmic reticulum [48]. From a biomechanical point of view, Schedel et al. [55] investigated the biomechanical source of CrM loading on sprint performance and found that athletes taking Cr had higher step frequencies. This was linked to the intense intracellular PCr ability to minimize muscle contraction and relaxation [55].

A faster resynthesis of PCr might explain a faster recovery during repeated bouts of high-intensity exercise [2]. However, despite the improvements of TD and BD in the present study, Cr supplementation did not improve fatigue index and RPE compared to PL. These results are supported by previous ones which showed unchanged FI [56] and perceived exertion [56,57] after Cr loading. However, our results contrasted those reported by Ateş, Keskin, and Bayraktar [27], which showed that 0.3 gr/kg of CrM loading over five days sped up recovery and decreased the losses between repeated sprint times, resulting in decreased FI. The difference in dosing procedures (relative vs. absolute) and target population may explain these discrepancies. FI tends to be more affected when performing an exhaustive exercise [58]. However, considering the RPE average values under both CrM and PL conditions (i.e., 6 points), values indicate that the 5mSRT in the present study was not too exhaustive for the performers. These results suggest that greater performance can be achieved with similar perceived exertion and FI after seven days of creatine loading.

Beyond improving physical performance, Cr has been shown to reduce mental fatigue and positively affect mood states, enhancing both recovery and performance [11,12]. Specifically, Cr supplementation has been associated with enhanced mood, reduced signs of depression, lower weariness, and more vitality, all of which contribute to improved psychological wellness [17,59]. These ergogenic actions were attributed to increased ATP availability, which may enhance the functioning of mood-regulating regions such as the prefrontal cortex and limbic system [17,60]. In the present study, CrM reduced perceived muscle soreness assessed using the Hooper questionnaire. An increase in PCr stores through Cr supplementation could stabilize muscle membranes, subsequently reducing loss in muscle function and attenuating intramuscular inflammation [61]. However, no other benefits were recorded regarding the affective valence and the other Hooper subscales under CrM compared to PL. Thus, supporting what has been documented by Furtado, Oliveira, Pereira, Veiga, Silva, and Abreu [56], the beneficial effect of CrM on physical performance in the present study cannot simply be attributed to a change in psychological state.

CrM supplementation may enhance cognitive functioning, particularly under conditions associated with reduced brain creatine availability, such as acute physiological or psychological stressors including intense exercise or sleep deprivation [12]. In the present study, attention, cognitive processing speed, and executive functioning assessed using the DCT showed improved cognitive performance following 7 days of CrM intake in well-rested male subjects. This outcome supports prior observations that short-term creatine CrM supplementation can positively influence cognitive performance when assessed after strenuous physical activity [11]. Similarly, Van Cutsem, Roelands, Pluym, Tassignon, Verschueren, De Pauw, and Meeusen [20] demonstrated that 20 g/day CrM across 7 days improved accuracy during a 90 min Stroop task. Furthermore, when concurrently experiencing a physiological stressor, the benefits of Cr supplementation may be more pronounced. For example, 20 g/day of CrM for 7 days improved multiple aspects of cognition during exposure to hypoxia (10% oxygen) in healthy individuals [25]. Taken together, the evidence suggests that creatine supplementation can enhance performance on tasks requiring sustained cognitive effort and may also mitigate perceptions of mental fatigue, even when stress or fatigue levels are low. Given its potential to enhance not only physical performance but also sleep quality and cognitive function, short-term creatine monohydrate loading may be a valuable strategy for athletes and active individuals seeking to optimize recovery and readiness during periods of intense training or competition.

While many studies focused on physical performance under different stressors conditions (e.g., sleep deprivation, hypoxia), the effects of CrM on sleep metrics, physical performances, and associated psycho-cognitive responses and recovery in well-rested healthy subjects are in an early stage. Therefore, the present study is the first to assess the effect of CrM loading on these performance aspects. However, this investigation suffers from various limitations that should be acknowledged. In fact, one major drawback is the lack of a pre-supplementation performance test. Although this may be considered a limitation in detecting changes over time, this issue is reduced by our use of a randomized, double-blind, crossover design in which every participant receives both the creatine and placebo supplements. We were able to effectively account for inter-individual variability in baseline performance, training status, and physiological parameters by comparing performance results upon completion of each condition in the same individuals. In addition, the precise biological pathways through which CrM affects outcomes are not fully understood, pointing to a clear need for more detailed biochemical and neurophysiological research. Moreover, actigraphy is a common method for measuring sleep patterns, but it may not capture all dimensions of sleep quality, such as sleep architecture or subjective feelings of restfulness. Also, muscle and brain creatine levels were not evaluated before and after the supplementation period. Thus, we could not determine the magnitude of the increase in tissue creatine. Furthermore, the effects of CrM may vary significantly between users, and findings from one population may not be generalized to another.

## 5. Conclusions

This study demonstrated that a short-term creatine monohydrate loading protocol (20 g/day for 7 days) improved subjective sleep quality during the supplementation phase, enhanced cognitive performance, and increased physical output during high-intensity intermittent exercise. Creatine monohydrate also reduced muscle soreness, but did not significantly affect objective sleep parameters, or recovery markers up to 72 h post-exercise. Therefore, the influence of creatine monohydrate on sleep appears to be limited to perceptual improvements rather than broad physiological changes. Given its potential to enhance not only physical performance but also sleep quality and cognitive function, short-term creatine monohydrate loading may be a valuable strategy for individuals seeking to optimize recovery and readiness during periods of intense training or competition. Clinicians and sports nutritionists could consider incorporating creatine monohydrate supplementation into recovery strategies for athletes facing sleep disturbances and cognitive performance challenges. The documented ergogenic potential suggests that short-term creatine loading may be a practical strategy to support performance and recovery in athletic settings, particularly when rapid benefits are desired. Considering the growing evidence of creatine’s dual impact on cognitive and physical performance, the development of clear guidelines emphasizing safe and evidence-based supplementation practices would be beneficial for athletic populations.

## Figures and Tables

**Table 1:** Actigraphy-derived sleep parameters recorded after 7 days of creatine monohydrate (CrM; 20 g/day) or placebo (PL) supplementation in physically active men (n = 14). Values are represented as mean ± SD for normally distributed variables (†) or median (IQR) for non-normally distributed variables (#).

Variable	Statistic	CrM	PL
In-bed time (hh:min:sec) #	MED/IQR	22:51:47 /00:18:35 *	23:07:24/00:16:30
Out-bed time (hh:min:sec) †	M (SD)	07:35:15 (00:41:37)	07:11:06 (00:45:38)
Total minutes in bed (min) †	M (SD)	488.65 (36.71)	471.65 (108.65)
Total sleep time (min) †	M (SD)	363.83 (34.14)	357.30 (90.87)
Sleep latency (min) †	M (SD)	30.68 (14.59)	25.09 (20.22)
Sleep efficiency (%) †	M (SD)	77.75 (5.40)	77.71 (5.97)

* Significantly different compared to placebo (*p* < 0.05); † normally distributed variables analyzed with the student *t*-test for dependent samples; # non-normally distributed variables analyzed with Wilcoxon signed-rank test; M: mean; SD: standard deviation; MED: median; IQR: interquartile; CrM: creatine monohydrate; PL: placebo.

**Table 2:** Subjective well-being and subjective sleep quality (SSQ) scores recorded after 7 days of creatine monohydrate (CrM; 20 g/day) or placebo (PL) supplementation in physically active men (n = 14). Measures include the Hooper questionnaire assessing sleep quality, stress, fatigue, and muscle soreness. Values are presented as mean ± SD for normally distributed variables (†) or median (IQR) for non-normally distributed variables #.

Variable	Statistic	CrM	PL
SSQ score †	M (SD)	7.43 (1.65) *	5.57 (1.79)
Hooper			
Sleep score #	MED/IQR	2/2.25	3/2.25
Stress score #	MED/IQR	1/1	2/2.25
Fatigue score †	M (SD)	2.93 (1.44)	3.14 (0.95)
Muscle soreness score †	M (SD)	2.93 (1.33) *	4.29 (1.38)
Index score †	M (SD)	9.36 (3.95)	10 (2.86)

* Significantly different compared to placebo (*p* < 0.05); † normally distributed variables analyzed with the student *t*-test for dependent samples; # non-normally distributed variables analyzed with Wilcoxon signed-rank test; M: mean; SD: standard deviation; MED: median; IQR: interquartile; CrM: creatine monohydrate; PL: placebo.

**Table 3:** Physical performance outcomes and perceived exertion recorded after 7 days of creatine monohydrate (CrM; 20 g/day) or placebo (PL) supplementation in physically active men (n = 14). Performance was assessed using the 5 m shuttle run test (5mSRT), including total distance (TD), best distance (BD), fatigue index (FI), and performance decrement (PD). Perceived exertion (RPE) was measured using the Borg CR-10 scale. Values are presented as mean ± SD for normally distributed variables (†) or median (IQR) for non-normally distributed variables (#).

Variable	Statistic	CrM	PL
TD (m) #	MED/IQR	762.50/67.50 *	662.50/75.00
BD (m) #	MED/IQR	150.00/40.00 *	125.00/00.00
FI (%) †	M (SD)	6.64 (13.58)	4.07 (8.05)
PD (%) †	M (SD)	13.27 (15.86)	8.93 (10)
RPE score †	M (SD)	6.25 (0.92)	6.06 (1.13)

* Significantly different compared to placebo (*p* < 0.05); † normally distributed variables analyzed with the student *t*-test for dependent samples; # non-normally distributed variables analyzed with Wilcoxon signed-rank test; M: mean; SD: standard deviation; MED: median; IQR: interquartile; CrM: creatine monohydrate; PL: placebo; TD: total distance; BD: best distance; FI: fatigue index; PD: performance decrement; RPE: perceived exertion; a.u: arbitrary unit.

**Table 4:** Cognitive performance and affective valence recorded after 7 days of creatine monohydrate (CrM; 20 g/day) or placebo (PL) supplementation in physically active men (n = 14). Measures include the digit cancellation test (DCT) for attention and processing speed, and the feeling scale (FS) for emotional valence. Values are presented as mean ± SD for normally distributed variables (†) or median (IQR) for non-normally distributed variables #.

Variable	Statistic	CrM	PL
DCT score †	M (SD)	64.29 (9.19) *	55.07 (8.33)
FS score #	MED/IQR	2/2.25	1/3

* Significantly different compared to placebo (*p* < 0.05); † normally distributed variables analyzed with the student *t*-test for dependent samples; # non-normally distributed variables analyzed with Wilcoxon signed-rank test; M: mean; SD: standard deviation; MED: median; IQR: interquartile; CrM: creatine monohydrate; PL: placebo.

## Data Availability

The data presented in this study are available on request from the corresponding author due to ongoing analyses and the need to preserve the integrity of planned follow-up studies.

## Supplementary Materials

The following supporting information can be downloaded at https://www.mdpi.com/article/10.3390/nu17243831/s1, Table S1: CONSORT checklist of the study.

## Abbreviations

The following abbreviations are used in this manuscript:

CrM	Creatine monohydrate
PL	Placebo
5mSRT	Five-meter shuttle run test
TD	Total distance
BD	Best distance
PD	Performance decrement
FI	Fatigue index
RPE	Rating of perceived exertion
SSQ	Sleep subjective quality
DCT	Digit cancellation test
FS	Feeling scale
PRS	Perceived recovery scale
DOMS	Delayed onset muscle soreness
CONSORT	Consolidated Standards of Reporting Trials
